# Exploring the Potential of Biochar Derived from Chinese Herbal Medicine Residue for Efficient Removal of Norfloxacin

**DOI:** 10.3390/molecules29092063

**Published:** 2024-04-29

**Authors:** Pengwei Li, Ziheng Zhao, Miaomiao Zhang, Hang Su, Ting Zhao, Weisheng Feng, Zhijuan Zhang

**Affiliations:** 1 College of Pharmacy, Henan University of Chinese Medicine, Zhengzhou 450046, China; lipwzz@hactcm.edu.cn (P.L.); 2021003010@hactcm.edu.cn (Z.Z.); 2022703100@hactcm.edu.cn (M.Z.); suhang@hactcm.edu.cn (H.S.); 2023703095@hactcm.edu.cn (T.Z.); fwsh@hactcm.edu.cn (W.F.); 2 Institute of Mass Spectrometer and Atmospheric Environment, Jinan University, Guangzhou 510632, China

**Keywords:** biochar, Chinese herbal residue, carbonization, adsorption, norfloxacin (NOR), response surface modeling (RSM)

## Abstract

One-step carbonization was explored to prepare biochar using the residue of a traditional Chinese herbal medicine, *Atropa belladonna* L. (ABL), as the raw material. The resulting biochar, known as ABLB4, was evaluated for its potential as a sustainable material for norfloxacin (NOR) adsorption in water. Subsequently, a comprehensive analysis of adsorption isotherms, kinetics, and thermodynamics was conducted through batch adsorption experiments. The maximum calculated NOR adsorption capacity was 252.0 mg/g at 298 K, and the spontaneous and exothermic adsorption of NOR on ABLB4 could be better suited to a pseudo-first-order kinetic model and Langmuir model. The adsorption process observed is influenced by pore diffusion, π–π interaction, electrostatic interaction, and hydrogen bonding between ABLB4 and NOR molecules. Moreover, the utilization of response surface modeling (RSM) facilitated the optimization of the removal efficiency of NOR, yielding a maximum removal rate of 97.4% at a temperature of 304.8 K, an initial concentration of 67.1 mg/L, and a pH of 7.4. Furthermore, the biochar demonstrated favorable economic advantages, with a payback of 852.5 USD/t. More importantly, even after undergoing five cycles, ABLB4 exhibited a consistently high NOR removal rate, indicating its significant potential for application in NOR adsorption.

## 1. Introduction

As a third-generation quinolone antibiotic, norfloxacin (NOR) has the characteristics of broad-spectrum antibacterial activity, strong anti-bacterial properties, and small toxic side effects, and is commonly used in medicine and aquaculture [1]. Quinolones are detected frequently in different settings [2]. In China, quinolones have been detected in hospital wastewater at levels ranging from 0.3 to 29 g/day [3]. Residues of NOR were found in livestock fecal matter at levels ranging from 1.89 to 225 mg/kg [4]. As a result of their infiltration into agricultural ecosystems via sewage, medical waste, and aquaculture practices, residual antibiotics accumulate in soil and water [5]. Consequently, they pose a significant threat to human health by entering the body through the food chain [6]. Furthermore, even low levels of antibiotics in soil can foster the emergence and dissemination of highly resistant bacteria, ultimately contributing to the development of antibiotic resistance in humans [7]. The elimination of antibiotics from wastewater is therefore imperative before the water can be discharged into the ecosystem [8].

A number of technologies are currently available for NOR removal, including biological treatment [9], advanced oxidation [10], and physical treatment [11]. Among these methods, physical treatment, particularly adsorption, is regarded as a highly effective approach for addressing antibiotics in wastewater due to its efficiency, simplicity, cost-effectiveness, and resistance to toxicity [12]. However, it is crucial to recognize that the adsorption efficiency is contingent upon the specific adsorbent type and its inherent characteristics [13,14,15]. To date, various adsorbents, including activated carbon [16], nanotube [17], metal–organic frameworks (MOFs) [18], osmotic membrane bioreactors [19], clay [20], and silica [21], have been extensively utilized for the removal of antibiotics. Specifically, biochar has attracted significant attention in the removal process of antibiotics owing to its easy availability, large surface area, abundance of surface functional groups, and developed pores [22,23]. Cheng et al. [24] developed a functionalized biochar for the adsorption of quinolone antibiotics from wastewater with a maximum adsorption capacity of up to 4.23 mg/g of NOR at room temperature. Luo et al. [25] investigated the preparation of ammonia-modified cassava pomace biochar with an adsorption capacity of 1.97 mg/g of NOR at 298 K. Unfortunately, the drawbacks of these adsorbents are that they may cause secondary pollution and are expensive or difficult to obtain locally [26], making them impractical and economically inefficient for water purification purposes [27]. In this study, a simple one-step carbonization method using locally available Chinese herbal medicine as raw material was used to prepare activated carbon that has a high adsorption capacity, and is environmentally friendly and cost-effective, which avoids problems such as environmental pollution due to the addition of chemical activators during the preparation and use of activated carbon. The prepared biochar demonstrates exceptional adsorption performance for NOR and displays notable adsorption selectivity for NOR when added to wastewater containing common anions and cations, but further adsorption studies in actual NOR-containing wastewater are needed.

The extensive utilization and widespread application of Chinese herbal medicine (CHM) globally have resulted in the accumulation of substantial residues, making it the most abundant waste at present [28]. It is reported that China generates approximately 70 million tons of CHM residues (CHMRs) annually [29], rendering it one of the most substantial biomass resources. *Atropa belladonna* L. (ABL), a well-known CHM, possesses high levels of lignin, cellulose, and hemicellulose, which encompass various functional groups, including carboxyl and hydroxyl groups [30]. As a result, biochar derived from ABL residues can serve as a viable alternative adsorbent with commendable chemical stability and mechanical robustness [31].

Herein, we have innovatively employed a one-step carbonization process to directly synthesize ABL biochar (ABLB) and subsequently examined its efficacy as an adsorbent for the elimination of NOR in a comprehensive manner. Moreover, response surface modeling (RSM) was employed to maximize the adsorption performance of NOR on ABLB. Additionally, the potential adsorption mechanisms of NOR on ABLB were also discussed, as well as its industrial economic feasibility. It is believed that this work will promote the comprehensive utilization of Chinese herbal medicine residues and guide the treatment of actual wastewater containing NOR.

## 2. Results and Discussion

### 2.1. Characterization

**Figure 1:**
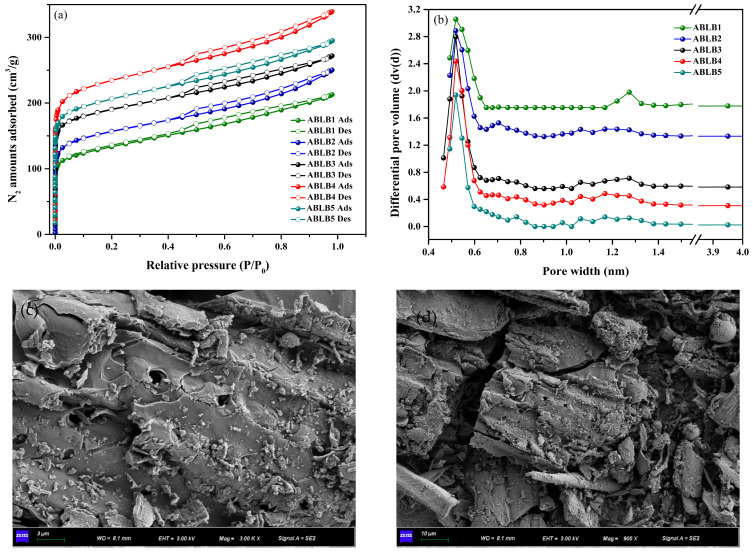
(**a**) N_2_ adsorption–desorption isotherms at 77 K; (**b**) The pore size distribution based on nonlocal density functional theory (NLDFT); (**c**,**d**) SEM images of ABLB biochar materials.

The N_2_ adsorption–desorption isotherms of biochar materials at 77 K are illustrated in Figure 1a. All biochar samples presented a composite adsorption isotherm with a combination of type I and type IV, suggesting the presence of predominant micropores [32]. As the carbonization time increased, the N_2_ adsorption capacity of biochar materials initially increased and then decreased, reaching the highest for ABLB4 biochar (ABL residue pyrolyzed directly at 1173 K under N_2_ flow in a tube furnace over 4 h). In the event of prolonged carbonization, the internal structure might collapse [33], leading to pore structure deformation. As depicted in Figure 1b, the pore size distribution of all samples primarily ranged from 0.4 to 1.3 nm, aligning with the findings of the N_2_ adsorption–desorption isotherms.

The BET surface area and pore volume of the biochar exhibited an initial increase followed by a decrease as the carbonization time was prolonged (Table 1). Notably, the ABLB4 sample displayed the highest specific surface area (S_BET_ = 812 m^2^/g) and total pore volume (0.50 cm^3^/g), indicating that a moderate extension of carbonization time is advantageous for achieving an improved porous structure under the same carbonization conditions. Thus, ABLB4 biochar was chosen for further adsorption experiments to explore its adsorption performance and mechanism.

The microscopic morphology of the as-synthesized ABLB4 was observed using SEM (Figure 1c,d). Obviously, the obtained biochar through high-temperature carbonization without the use of chemical reagents preserved a significant amount of plant-specific fiber structure. The existence of numerous small particles on the surface of ABLB4 can be attributed to the degradation of cellulose and other macromolecular organic matter during pyrolysis [34]. SEM-EDX microphotographs of ABLB4 revealed a uniform distribution of elements such as N and O on the biochar, facilitating the provision of numerous adsorption sites for NOR adsorption (Figure S6). Moreover, the cellulose and lignin contents of the ABL residues were determined to be 40.6% and 20.8%, respectively, which were reconstituted into graphite microcrystalline and amorphous bodies in biochar in the pyrolysis process [35], indicating significant potential for utilization as a biochar adsorbent.

The obtained biochar materials exhibited a decrease in H and O content, while C content showed a substantial increase compared to the ABLB materials (Table 2). This suggests that decomposition and cracking occurred during the process of dehydration and decarboxylation [36]. The elemental composition of biochar can serve as an indicator of the presence of groups to a certain degree. Following high-temperature carbonization, the evaporation of surface water from biochar results in a reduction in the proportions of H and O, and an increase in the proportion of C as observed in elemental analysis. Thus, the formation of a ring-closed conjugate system becomes more feasible, leading to a decrease in system energy and an increase in aromaticity. As the proportions of H and O diminish during the carbonization process, the formation of hydrogen bonds becomes increasingly challenging in the system, which is manifested by the decrease in hydrophilicity of the biochar. The lower values of H/C, O/C, and (N + O)/C demonstrated that the ABLB4 material had a high degree of aromaticity and a hydrophilic surface. Interestingly, the H/C, O/C, and (O + N)/C values of the ABL biochar materials were found to be similar, indicating similar degrees of aromaticity and polarity [37]. These findings indicate that altering the carbonization time does not significantly impact the element content and polarity of ABL biochar samples at a consistent carbonization temperature.

All ABL biochar samples exhibit favorable thermal stability, particularly ABLB4, as depicted in Figure S2. Nevertheless, when the carbonization time is extended to 5 h, the thermal stability of the material may drop sharply at about 400 °C due to the collapse of the internal structure, which confirms the infeasibility of excessive carbonization [38,39].

### 2.2. Adsorption Performance

#### 2.2.1. Effect of Adsorbent Dosage

**Figure 2:**
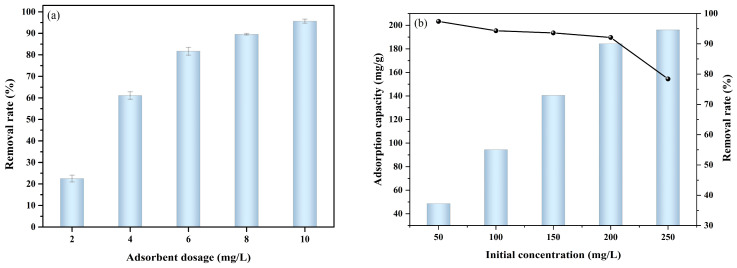

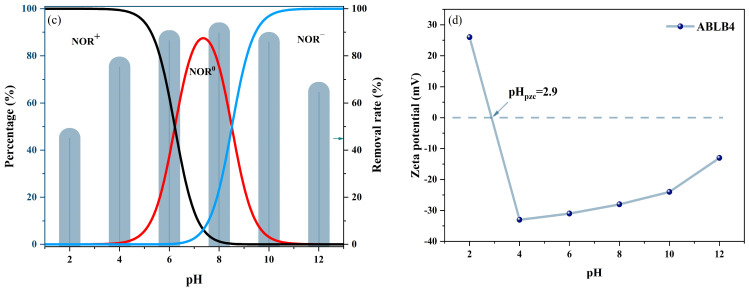
(**a**) Impact of biochar dosage on the removal rate of NOR. (**b**) Influence of initial concentration on NOR adsorption. (**c**) Influence of pH value on the removal rate of NOR (initial concentration = 200 mg/L, adsorbent dosage = 10 mg, and contact time = 1440 min). (**d**) Zeta potential of ABLB4 (adsorbent = 1000 mg/L, T = 298 K).

The impact of biochar dosage on the removal efficiency of NOR is depicted in Figure 2a. It is observed that as the adsorbent dosage increases, the removal rate of NOR gradually improves. The NOR removal was maximized at pH = 7 with an ABLB dosage of 10 mg. This finding indicates that the optimal amount of adsorbent dosage ensures a high removal rate of NOR and low costs.

#### 2.2.2. Effect of Initial NOR Concentration

Figure 2b illustrates the impact of initial NOR concentration on the adsorption performance of ABLB4. When pH = 7 and the initial concentrations ranged from 50 to 200 mg/L, the removal efficiencies of NOR exceeded 96%. Interestingly, with a further increase in initial NOR concentration (250 mg/L), the removal rate decreased to 78%, while the adsorption capacity of NOR was not significantly increased. This decrease in removal rate may be ascribed to the adsorption sites of ABLB4 in the solution reaching saturation at a higher concentration of NOR, implying that the optimal initial concentration of NOR for subsequent adsorption experiments is 200 mg/L.

#### 2.2.3. Effect of Solution pH

Most notably, the removal efficiency of NOR on biochar is significantly influenced by the initial solution pH, as it can affect the structure of NOR and the surface chemistry of the adsorbent (Figure 2c). The point of zero charge (pH_pzc_) was assessed to characterize the electrochemical state of the adsorbent surfaces in the solution (Figure 2d) [40,41]. It is evident that within the pH range of 2 to 8, the adsorption efficiency of NOR on ABLB4 is progressively enhanced due to the electrostatic attraction, resulting in a maximum removal rate exceeding 90%. In contrast, the removal rate of NOR decreases substantially when the pH value exceeds eight. This decrease can be attributed, in part, to the ionization of NOR (pKa = 6.22 and 8.51) into NOR^−^ under such conditions, leading to electrostatic repulsion with the negatively charged carbon. Despite variations in NOR removal efficiency at different pH values, the wide pH range of 2–12 still allows for the practical application of ABLB4 in real water bodies.

#### 2.2.4. Adsorption Kinetics

**Figure 3:**
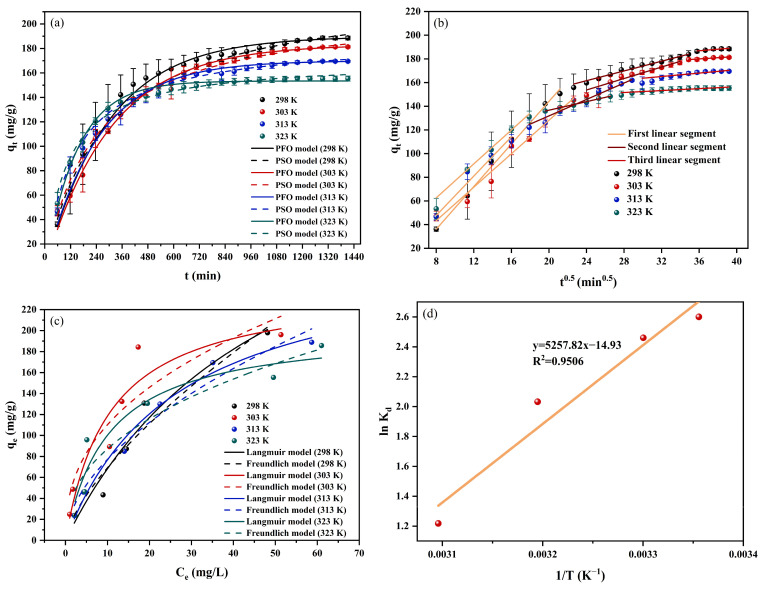
(**a**) PFO and PSO adsorption kinetics models of NOR adsorption by ABLB4; (**b**) Weber–Morris intraparticle diffusion model of NOR adsorption by ABLB4; (**c**) Experimental and fitted adsorption isotherms of NOR on ABLB4; (**d**) Thermodynamics plots for adsorption of NOR on ABLB4.

The investigation of kinetic studies is crucial for comprehending the adsorption performance in relation to the rate constant order. To assess the adsorption kinetics of NOR on ABLB4, various models including PFO, PSO, and IPD used the experimental data at different temperatures. The outcomes of these analyses are depicted in Figure 3a,b, and the corresponding kinetic parameters are listed in Tables S2 and S3.

Evidently, the PFO kinetic model exhibited better matching in describing the adsorption kinetics of all samples compared to the PSO kinetic model, as indicated by generally higher R^2^. Furthermore, the q_e_ values determined through the PFO model exhibited a higher degree of agreement with experimental results, indicating the suitability of the PFO model. Additionally, higher temperatures expedite the kinetic process, leading to the attainment of adsorption equilibrium within 480 min at 323 K.

Furthermore, the Weber–Morris intraparticle diffusion model was employed to offer a more thorough explanation of the adsorption mechanism of NOR on ABLB4. As depicted in Figure 3b, the adsorption process of NOR can be segmented into three distinct stages. The initial stage involves film diffusion, during which NOR moves towards the external surface of ABLB4 from the boundary layer through rapid diffusion, resulting in a substantial increase in NOR adsorption. The Ci value of ABLB4 (35.80 mg/g) at this stage signifies a significant diffusion impact within the boundary layer (Table S3). During the intra-particle diffusion stage, NOR molecules permeate gradually into the pore structure of ABLB4, ultimately reaching equilibrium. Despite the occurrence of intra-particle diffusion of NOR molecules, it is noted that the IPD model fitting curve does not intersect with the origin, indicating that IPD is not the sole limiting factor in NOR adsorption [42].

#### 2.2.5. Adsorption Isotherm

To further understand the NOR adsorption behavior on ABLB4 at various temperatures, the Langmuir, Freundlich, and Temkin isotherm models were utilized (Figure 3c and Figure S7). As listed in Table 3, the R^2^ values for the Langmuir model were consistently higher than those for the Freundlich model, indicating that the Langmuir model is more appropriate for describing the adsorption behavior of NOR on ABLB4. In assessing the adsorption performance of NOR, the *K_L_* value is commonly employed. A *K_L_* value between 0 and 1 signifies excellent adsorption performance. The obtained *K_L_* values for all temperatures fall within this range, implying that the adsorption of NOR molecules on the ABLB4 sample is favorable. Significantly, the *n* value fell within the range of 2.17–2.48, indicating favorable physical processes, while the reciprocal of *n* (1/*n*) ranged from 0.46 to 0.54, suggesting a high degree of heterogeneity in ABLB4 [43]. The values of parameter b in the Temkin model were all greater than 20 J/mol, indicating the presence of chemisorption in the adsorption process. Additionally, the Langmuir model was employed to calculate the maximum adsorption capacity of NOR on the ABLB4 sample at 298 K, resulting in a value of 252.0 mg/g. As shown in Table S4, the adsorption performance of ABLB4 is outstanding among reported adsorbents for NOR adsorption.

#### 2.2.6. Thermodynamic Analysis

The calculated thermodynamic parameters were plotted against 1/T, as illustrated in Figure 3d and Table S5. Clearly, the negative Δ*G* values for ABLB4 indicate that the adsorption of NOR on ABLB4 was spontaneous. In addition, it is noted that the absolute value of Δ*G* decreases with increasing temperature, implying that higher temperatures hinder the adsorption process. Moreover, the negative value of Δ*H* (−43.79 KJ/mol) suggests that the NOR adsorption on ABLB4 is exothermic [44].

### 2.3. Adsorption Mechanism

Based on the fitting results of the adsorption kinetics, it appears that ABLB4 and NOR interactions are dominated by the physical adsorption process. Hence, a combination of zeta potential, XRD, FTIR, and XPS characterization techniques was employed to investigate the adsorption mechanisms, including pore filling, electrostatic interactions, hydrogen bonding, and π–π stacking.

The ABLB4 material exhibits a significant presence of micropore and mesopore structures. According to the IPD model, it is evident that the initial stage of NOR attachment to the ABLB4 surface occurs rapidly and in large quantities, while the subsequent stage involves the penetration of NOR molecules into the internal pores of ABLB4 through intra-particle diffusion. This observation highlights the crucial role of pore filling in the adsorption process of NOR on ABLB4.

The analysis of zeta potential and pH effects demonstrates that the adsorption of NOR on ABLB4 is significantly influenced by electrostatic interactions. This was confirmed through time-dependent UV full-wavelength scans and experiments at varying concentrations, which demonstrated that NOR did not undergo decomposition or rearrangement over time or concentration (Figure S8). The results indicated that the observed NOR adsorption is indeed a result of electrostatic interactions in the biochar-loaded solutions, rather than photocatalytic degradation or rearrangement of NOR [45]. It is clear that the pH_pzc_ for ABLB4 is 2.9, indicating the presence of acidic functional groups on the surface of the biochar. Furthermore, NOR possesses two dissociation constants with corresponding pK_a_ values of 6.22 and 8.51. As a result, NOR primarily exists as a cationic species when the pH is below 6.22. The zwitterionic form of NOR can be observed within a pH range of 6.22 to 8.51, while the anionic form is present when the pH exceeds 8.51 [46]. Hence, when the pH of the solution surpasses the pH_pzc_ (2.9) but remains within the range of 2.9–8.51, the negatively charged ABLB4 demonstrates a strong electrostatic interaction with both NOR^+^ and NOR. Conversely, when the pH value exceeds 8.51, the affinity between the negatively charged ABLB4 and NOR^−^ weakens, resulting in a decrease in NOR removal efficiency.

**Figure 4:**
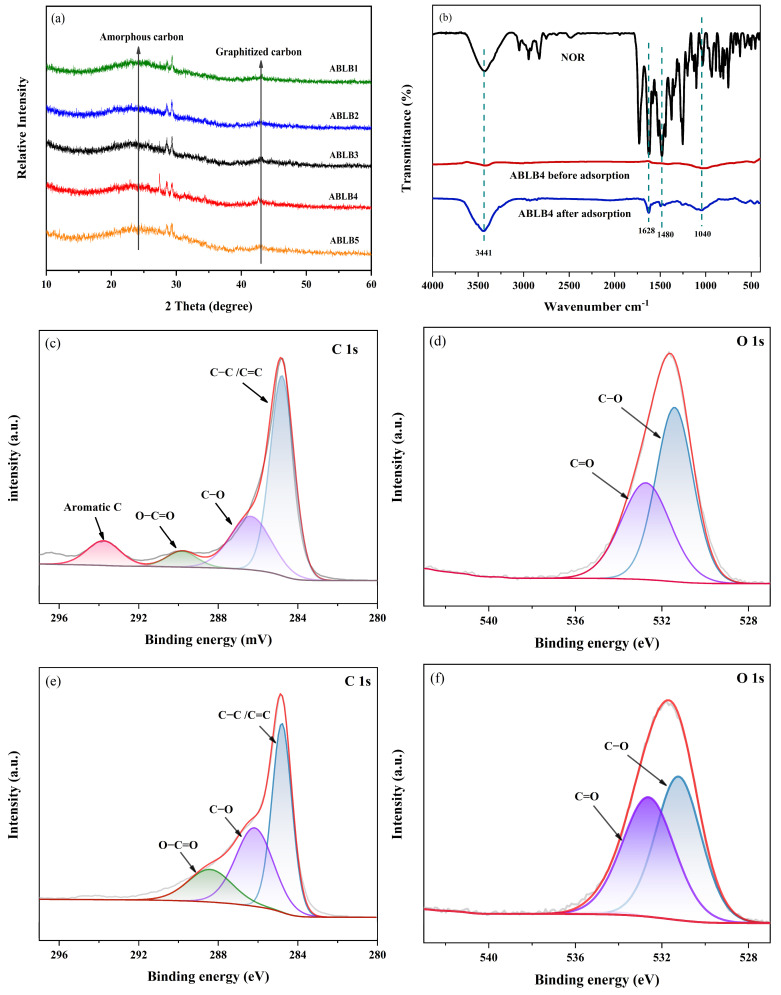
(**a**) XRD patterns of ABL biochar. (**b**) FTIR spectra of ABLB4 and NOR. (**c**) C 1s and (**d**) O 1s XPS analysis of ABLB4. (**e**) C 1s and (**f**) O 1s XPS analysis of ABLB4 after NOR adsorption.

The XRD patterns of prepared ABL biochar exhibit wide reflections at 24° and 44° corresponding to (002) and (100) planes, respectively, indicating the presence of amorphous biochar resulting from the accumulation of an aromatic layer structure [47]. The peaks observed at 2θ (25°–30°) suggest a disordered layered structure of SiO_2_, potentially attributed to the presence of a Si element within the sample itself, as illustrated in Figure 4a and documented in Table S8 [48].

To explore the adsorption mechanism, the ABLB4 sample was characterized with FTIR (Figure 4b). The peak observed at 1480 cm^−1^ is attributed to the stretching vibration of the C–O and C–H structures present in the alkene [49,50]. Similarly, the peak at 1040 cm^−1^ is assigned to the expansion and contraction of the C–O–C structure [51]. The FTIR spectrum of NOR revealed a prominent vibrational peak at 3433 cm^−1^, suggesting that the observed increase in peak intensity at 3441 cm^−1^ during adsorption may be attributed to the O–H vibration within the NOR molecule. The blue shift in the O–H position after adsorption implies a potential interaction between biochar and the oxygen-containing functional groups of the NOR molecule, potentially forming hydrogen bonds [52]. The NOR molecule exhibits a nearly planar structure, consisting of a benzene ring and a six-membered ring. The F group attached to the benzene ring carries a negative charge and possesses a strong electron-withdrawing ability, resulting in a reduction in electron density on the benzene ring and its function as an electron acceptor. Hence, the π–π interaction occurs between the π–electron donor present on the adsorbent and the π–electron acceptor on the NOR molecule. Furthermore, the hydroxyl group on the adsorbent acts as an electron donor, while the aromatic ring on the adsorbent serves as a potent electron donor capable of engaging in π–π interaction with the electron acceptor on the NOR. The significant enhancement in the absorption peak within the approximately 1700–1500 cm^−1^ was observed after adsorption, which may be associated with the formation of π–π stacking between the organic ligand in ABLB4 and the benzene rings of NOR [53]. This π–π bonding is an important mechanism for NOR adsorption to ABLB4 [54].

**Figure 5:**
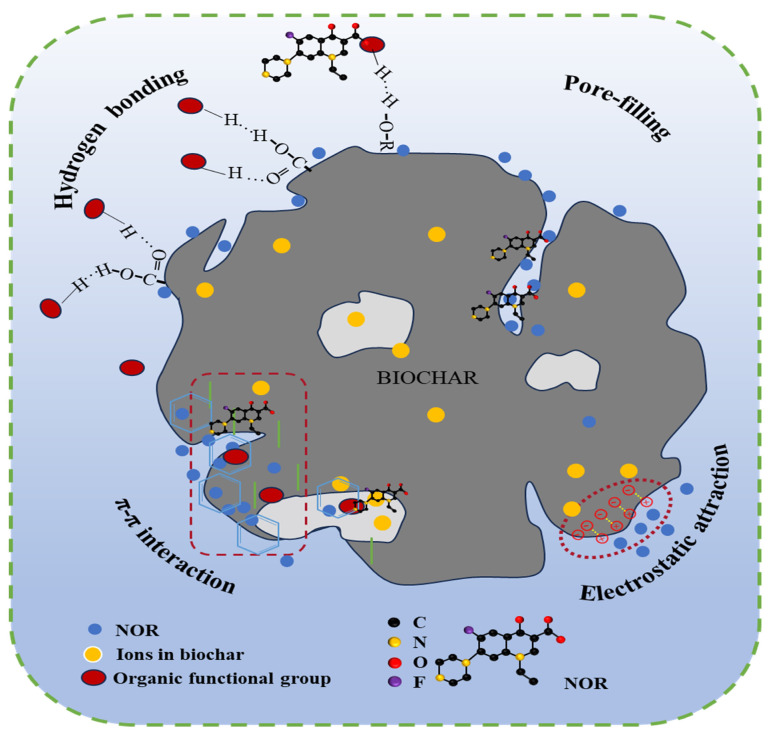
The NOR adsorption mechanisms of ABLB4.

Figure 4c–f shows the XPS of ABLB4. The C 1s of the ABLB4 sample was divided into four peaks at 284.8, 286.3, 289.8, and 293.7 eV, corresponding to C–C, C–O, O–C=O, and π–π*, respectively [55], indicating that there are functional groups in the structure that can generate π–π interactions and provide more active sites for subsequent adsorption, and, at the same time, some satellite peaks are found around 293.7 eV [56]. The O 1s spectrum of the ABLB4 sample was divided into two peaks at a binding energy of 531.4 and 532.7 eV, corresponding to C=O and OH, respectively. This is coincident with the finding by the FTIR analysis of the biochar structure. To further explore the mechanism of NOR adsorption on biochar, the adsorbed ABLB4 samples were analyzed, as shown in Figure 4d,e; the change in the C 1s peak of the ABLB4 sample after NOR adsorption indicates that the benzene ring structure and C=O bond as an electron donor group participate in the adsorption process, which provides a way to explore the specific mechanism of NOR adsorption [57]. As discussed above, Figure 5 summarizes the NOR adsorption mechanisms of ABLB4.

### 2.4. Industrial Economic Feasibility Analysis

As shown in Figure S3, the ABLB4 sample and purchased biochar for commercial wastewater treatment were tested in the same environment. The removal efficiency of NOR measured by commercial biochar was only 4% [58], while the removal rate of NOR by ABLB4 was as high as 97%. As listed in Table S6, clearly, based on energy consumption for our research, ABLB4 prepared using the green method had a greater payback value than commercial biochar, suggesting that it has great application prospects in practical production.

### 2.5. Response Surface Method (RSM) Regression Analysis

To optimize the NOR removal rate conditions, an RSM regression analysis was conducted based on the Box–Behnken design. In this study, the three variables selected were temperature (A), initial concentration (B), and pH value (C). In light of the experimental NOR adsorption data, the regression equation was developed, as presented in Table 4.

Table 4 lists the significance of the quadratic regression model assessed through the examination of *F*-values, *p*-values, and R^2^. The R^2^ value indicates a high level of fit (0.9204), and the predicted maximum adsorption capacity (178.5 mg/g) closely aligns with the experimental value (192.4 mg/g), suggesting that the model’s predictive capability is in agreement with the experimental findings. The high *F*-values (9) and low *p*-values (<0.05) indicate that the model parameters have a significant impact on the prediction of response variables. The initial concentration was found to be a statistically significant term based on its F and *p* values. Ideally, a signal-to-noise ratio greater than 4 is desired, and the signal-to-noise ratio of 9.12 observed in this study indicates that the signal is sufficient [59].

The NOR removal rate varied between 34.9% and 95.6% across different experiments. The contour plot analysis and cube optimization results for degradation efficiency are displayed in Figure S4e,f. The center point of the medium height line plot and the cube represents the maximum NOR removal rate.

Thus, the optimal conditions for a maximum NOR removal rate (97.4%) included a temperature of 304.8 K, an initial concentration of 67.1 mg/L, and a pH of 7.4. In this study, the optimized parameters describe the ability to remove NOR over a fairly wide concentration range (25–250 mg/L).

### 2.6. Influence of Other Coexisting Ions

**Figure 6:**
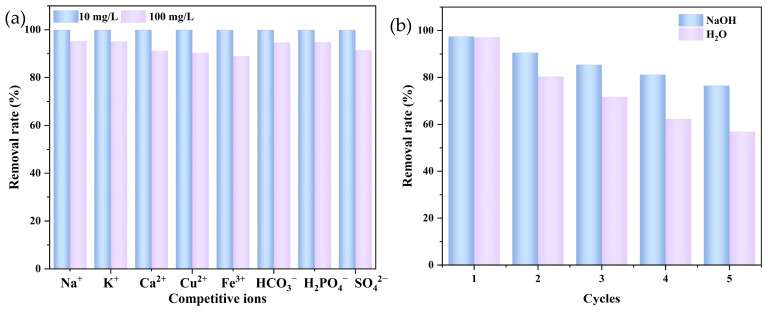
(**a**) Effects of co-existing competitive ions on NOR adsorption. (**b**) Five NOR adsorption-desorption cycles on ABLB4 with NaOH or ultrapure water as eluent.

In aqueous environments, different ions engage in competitive adsorption of NOR on the biochar ABLB4. Simulations indicated that ABLB4 exhibited a notable preference for the removal of NOR, with minimal interference from competing ions (Figure 6a). Nevertheless, when ion concentrations surpass 10 mg/L, the presence of Ca^2+^, Cu^2+^, and Fe^3+^ can slightly hinder NOR adsorption, particularly Cu^2+^ and Fe^3+^, as a result of competition for adsorption sites. Despite increased cation levels, the efficiency of NOR removal by ABLB4 remains above 90%, showing favorable selectivity.

### 2.7. Regeneration Ability of ABLB4 Biochar

The ability to regenerate is a crucial factor in evaluating the durability of an adsorbent. In this study, a NaOH solution with a concentration of 1 mol/L was employed to desorb the saturated ABLB4 biochar. After thorough washing and drying, the regenerated biochar was reused for adsorption of NOR to assess its reproducibility. As depicted in Figure 6b, the removal efficiency of NOR decreased only slightly to 90% in the second recovery cycle when NaOH was utilized as the eluent, and remained above 76% even after five cycles. Conversely, when ultrapure water was employed as the eluent for five cycles, the removal rate of NOR was no more than 56%. Therefore, NaOH was selected as the optimal eluent for the recycling of the ABLB4 biochar.

## 3. Materials and Methods

### 3.1. Chemicals and Materials

NOR (M_w_ = 319.33 g/mol; USP grade) was acquired from Shanghai Aladdin Biochemical Technology Co., Ltd. (China). The ABL residues were obtained in a Chinese pharmaceutical company in Henan, China. Meanwhile, the commercial activated carbon was produced from a biochar factory in the same province. No further processing was performed on the chemicals once they were received.

### 3.2. Synthesis of ABL Biochar

**Scheme 1:**
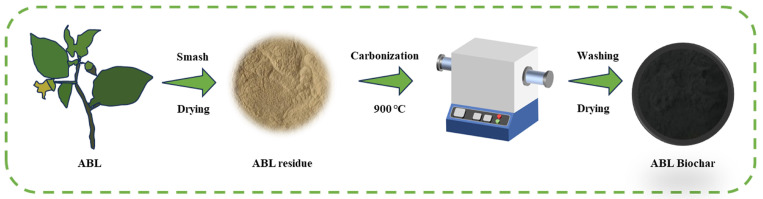
Flowchart of ABL biochar preparation.

The synthesis process can be described as Scheme 1. First, 2 g of water-washed, air-dried, and sieved ABL residues were pyrolyzed directly at 1173 K under N_2_ flow in a tube furnace over a period (1, 2, 3, 4, or 5 h) at a heating rate of 5 °C/min. Then, the synthesized biochar was designated as “ABLB1/2/3/4/5”. To obtain a neutral pH, the prepared samples were washed in distilled water, and then dried at 378 K for a duration of 12 h.

### 3.3. NOR Removal Study

In this study, an ultraviolet–visible (UV/VIS) double-beam spectrophotometer (Hitachi U-3900H, Tokyo, Japan) was used to analyze the NOR concentration at a wavelength of 273 nm [60]. Correspondingly, the adsorption capacities (q_e_, mg/g), removal rate (%), adsorption kinetics, isotherms, and thermodynamic parameters were calculated using the equations listed in Table S1.

#### 3.3.1. Adsorption Studies

By adding separately 2.0, 4.0, 6.0, 8.0, and 10 mg of adsorbents into flasks with NOR solution, the effect of adsorbent dosage on NOR uptake was examined. A NOR concentration range of 50–250 mg/L was observed in the first concentration test. In order to investigate the impact of contact time, the adsorption process was measured within 120 min. An adjustment of pH in the range of 2–12 was made to the solution to determine its effect on NOR adsorption. Adsorption experiments were conducted in triplicate for each sample.

#### 3.3.2. Adsorption Kinetics, Isotherms, and Thermodynamics

Firstly, 10 mg of ABLB4 was initially added to 10 mL NOR solution at a concentration of 200 mg/L for 1440 min at 298 K with aliquots taken continuously. Three separate models were used to analyze the adsorption kinetics of NOR, namely, the pseudo-first-order (PFO), pseudo-second-order (PSO), and intra-particle diffusion (IPD).

In isotherm experiments, NOR was initially concentrated at 50–250 mg/L, and temperatures were set at 298 K, 303 K, 313 K, and 323 K, respectively.

### 3.4. Reusability Performance

Each cycle, ABLB4 biochar with adsorbed NOR was regenerated by washing with 1 M of NaOH solution or water. The recovered biochar was then dried in an oven to investigate its reusability and stability.

### 3.5. Response Surface Method (RSM)

In this study, the initial concentration, temperature, and pH value were chosen as variables. The relationship between y (removal rate) and these encoded variables in RSM is described by an empirical quadratic polynomial, as presented in Equation (1).

(1) $$y=a_{0}+\sum a_{i}X_{i}+\sum a_{ij}X_{i}X_{j}+\sum a_{ii}{X_{i}}^{2}$$

where y is the predicted response (removal rate), i and j are the types of variables, X_i_ and X_j_ are the variables, a_0_ is the constant, a_i_ represents the linear coefficient, a_ij_ is the interaction coefficient, and a_ii_ represents the quadratic coefficient, respectively. Finally, to clarify the best adsorption conditions, multi-factor analysis of variance and quadratic regression were used to determine the influence of each variable on the removal rate [61,62].

## 4. Conclusions

In the present study, ABL residue biochar materials were directly prepared by one-step carbonization and used to treat NOR pollutants. Through the characterization of the pore structure (S_BET_ = 812 m^2^/g) and the functional groups on the surface, it was proved that ABLB4 exhibited great potential for NOR elimination. The calculated maximum NOR adsorption capacity by the Langmuir model is 252.0 mg/g at 298 K. And, the pseudo-first-order model is well suited to describe the adsorption kinetic data. Moreover, the adsorption of NOR onto ABLB4 took place spontaneously and exothermally. This whole adsorption process may be related to pore diffusion, π–π interaction, electrostatic interaction, and hydrogen bonding between ABLB4 and NOR molecules. Furthermore, the NOR adsorption performance is optimized using RSM, reaching a maximum removal rate (97.4%) with a temperature of 304.8 K, an initial concentration of 67.1 mg/L, and a pH of 7.4. In addition, the biochar material produced demonstrated favorable economic benefits, with a payback of 852.5 USD/t. As a result of these findings, we have gained a deeper understanding of the NOR adsorption behaviors and mechanisms on ABLB4 material, and advanced the utilization of biochar adsorbents in the elimination of antibiotics.

## Figures and Tables

**Table 1:** Parameters of porous structure for the synthesized biochar.

Samples	S_BET_ (m^2^/g)	S_Langmuir_ (m^2^/g)	V_Micro_ (cm^3^/g)	V_Total_ (cm^3^/g)	Average Pore Diameter (nm)
ABLB1	499	639	0.12	0.33	2.69
ABLB2	665	828	0.20	0.42	2.51
ABLB3	664	800	0.20	0.40	2.40
ABLB4	812	1002	0.24	0.50	2.40
ABLB5	712	882	0.21	0.43	2.42

**Table 2:** Elemental composition of prepared biochar.

Samples	C (%)	H (%)	O (%)	N (%)	S (%)	H/C	O/C	(O + N)/C
ABLB1	71.85	1.09	8.82	0.44	0.15	0.02	0.12	0.13
ABLB2	67.91	0.72	7.53	0.34	0.06	0.01	0.11	0.12
ABLB3	71.31	0.58	6.72	0.00	0.92	0.01	0.09	0.09
ABLB4	70.99	0.58	5.91	0.00	0.16	0.01	0.08	0.08
ABLB5	71.68	0.43	6.52	0.34	0.04	0.01	0.09	0.10

**Table 3:** Parameters for the adsorption isotherm model.

Temperature		Langmuir			Freundlich			Temkin		
	q_e,exp_ (mg/g)	q_e,cal_ (mg/g)	*K_L_* (L/mg)	R^2^	*K_F_* ((mg^1−n^ L^n^)/g)	*n*	R^2^	KT (L/g)	b (J/mol)	R^2^
298 K	192.4	251.97	0.07	0.98	33.94	2.17	0.93	0.44	41.44	0.8526
303 K	183.37	242.88	0.10	0.88	43.51	2.48	0.83	0.56	56.26	0.8654
313 K	164.28	220.58	0.06	0.99	28.91	2.22	0.95	0.60	50.79	0.9456
323 K	156.93	202.98	0.10	0.90	34.05	2.45	0.86	0.91	60.43	0.9132

**Table 4:** Parameters of variance (ANOVA) for the quadratic model.

Source	SS	DF	MS	*F*-Value	*p*-Value
Model	5769.83	9	641.09	9.00	0.0042
A-temperature (K)	6.25	1	6.25	0.088	0.7757
B-initial concentration (mg/L)	2191.22	1	2191.22	30.75	0.0009
C-pH	203.31	1	203.31	2.85	0.1351
AB	0.90	1	0.90	0.013	0.9136
AC	7.225 × 10^−3^	1	7.225 × 10^−3^	1.014 × 10^−3^	0.9922
BC	19.80	1	19.80	0.28	0.6144
A^2^	233.77	1	233.77	3.28	0.1130
B^2^	369.38	1	369.38	5.18	0.0569
C^2^	2781.28	1	2781.28	39.03	0.0004
Residual	498.83	7	71.26		
Lack of Fit	494.16	3	164.72	141.03	0.0002
Pure Error	4.67	4	1.17		
Cor Total	6268.66	16			
Removal rate (%) = 85.06 − 0.88 × A − 16.55 × B + 50.4 × C − 7.45 × A^2^ + 9.37 × B^2^ − 25.70 × C^2^					

Note: R^2^ = 0.9204, DF denotes degrees of freedom, SS represents the sum of squares, and MS signifies the mean square. *p* value < 0.05 indicates that the model terms are significant.

## Data Availability

Data will be made available on request.

## Supplementary Materials

The following supporting information can be downloaded at https://www.mdpi.com/article/10.3390/molecules29092063/s1, Figure S1: Effect of pH on the adsorption capacity of NOR; Figure S2: TGA curve of ABL biochar samples; Figure S3: NOR adsorption effect comparison of ABLB4 and commercial biochar; Figure S4: Response surface method (RSM) regression analysis; Figure S5: Full-wavelength scan of NOR; Figure S6: SEM-EDX microphotographs of ABLB4; Figure S7: Temkin isotherm of ABLB4; Figure S8: (a) UV full-wavelength scanning spectra of NOR with time (b) UV full- wavelength scanning spectra of NOR at different concentrations. Table S1: Detailed information on involved formulas and models; Table S2: Kinetic constants of pseudo-first-order and pseudo-second-order models; Table S3: Kinetic constants for the IPD model; Table S4: Lists of maximum NOR adsorption capacity on various biochar and other adsorption materials; Table S5: Thermodynamic parameters for NOR adsorption; Table S6: Energy consumption of biochar used in industrial wastewater production (USD/t); Table S7: Elemental composition of raw ABL residues; Table S8: ICP-OES analysis of raw ABL residues. References [59,63,64,65,66,67] are cited in the Supplementary Materials.
