# Supplementary material for: Exploring the Potential of Biochar Derived from Chinese Herbal Medicine Residue for Efficient Removal of Norfloxacin
Source: Molecules. 2024 Apr 29;29(9):2063. doi: 10.3390/molecules29092063 (PMC11085230; doi:10.3390/molecules29092063)
Supplement: Supplementary file 1 [file molecules-29-02063-s001.zip › molecules-2970236-supplementary.pdf]

***Supporting Information for***

# Exploring the Potential of Biochar Derived from Chinese Herbal Medicine Residue for Efficient Removal of Norfloxacin

Pengwei Li [1,§], Ziheng Zhao [1, §], Miaomiao Zhang [1], Hang Su [1], Ting Zhao [1], Weisheng Feng [1], Zhijuan Zhang [1, 2,*]

1. College of Pharmacy, Henan University of Chinese Medicine, Zhengzhou 450046, China

2. Institute of Mass Spectrometer and Atmospheric Environment, Jinan University, Guangzhou 510632, China

*Corresponding author, Email: tzjzhang@hactcm.edu.cn (Z.J. Zhang);

§ These authors contributed equally.

## Characterization

A physisorption analyzer (Autosorb $IQ_2$, Quantachrome) was used to determine the pore structures of ABL biochars. An elemental analyzer (EA, Elementar UNICUBE) was utilized to analyze the element composition. The scanning electronic microscopy (SEM, Zeiss Gemini 300) coupled with energy dispersive spectroscopy (EDS, Zeiss Gemini 300) was used to examine the surface morphology and chemical composition of biochar samples. A Fourier transform infrared spectrometer (FTIR, Nicolet iS5, Thermo Scientific) was used to determine the surface functional groups, while an X-ray photoelectron spectroscopy (XPS, Thermo-Scientific K-Alpha) was utilized to investigate their chemical compositions. By using an X-ray diffractometer (XRD, Bruker D8), the crystal structure was investigated. Finally, a thermogravimetric analyzer (TGA, TA550) was used to determine the thermal stability.

# FIGURE CAPTIONS

Figure S1 Effect of pH on the adsorption capacity of NOR.

Figure S2 TGA curve of ABL biochar samples.

Figure S3 NOR adsorption effect comparison of ABLB4 and commercial biochar.

Figure S4 Response surface method (RSM) regression analysis.

Figure S5 Full-wavelength scan of NOR.

Figure S6 SEM-EDX microphotographs of ABLB4.

Figure S7 Temkin isotherm of ABLB4.

Figure S8 UV full-wavelength scanning spectra of NOR with time. (b) UV full-wavelength scanning spectra of NOR at different concentration.

## TABLE CAPTIONS

Table S1 Detailed information of involved formulas and models.

Table S2 Kinetic constants of pseudo-first-order and pseudo-second-order models.

Table S3 Kinetic constants for the IPD model.

Table S4 Lists of maximum NOR adsorption capacity on various biochar and other adsorption materials.

Table S5 Thermodynamic parameters for NOR adsorption.

Table S6 Energy consumption of biochar used in industrial wastewater production ($/t).

Table S7 Elemental composition of raw ABL residues.

Table S8 ICP-OES analysis of raw ABL residues.

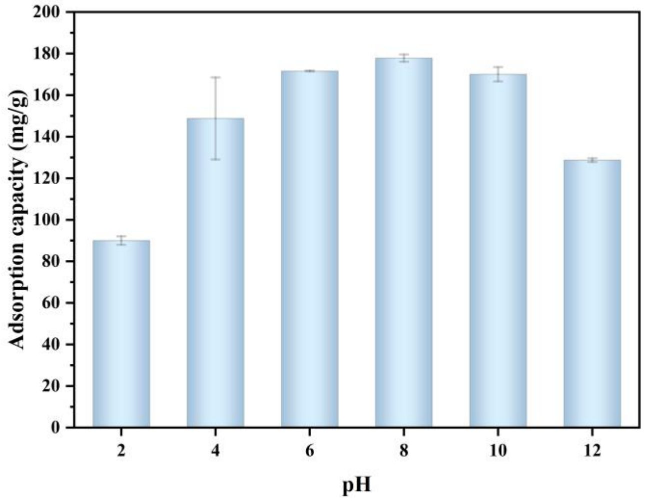


**Figure S1.** Effect of pH on the adsorption capacity of NOR (initial concentration 200 mg/L, adsorbent dosage 10 mg, contact time 1440 min).

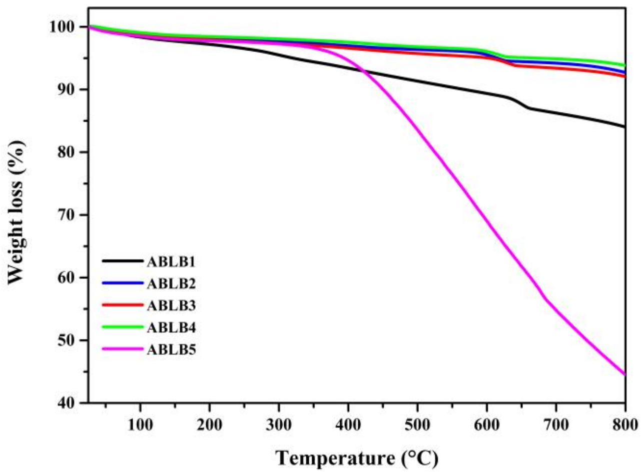


**Figure S2.** TGA curve of ABL biochar.

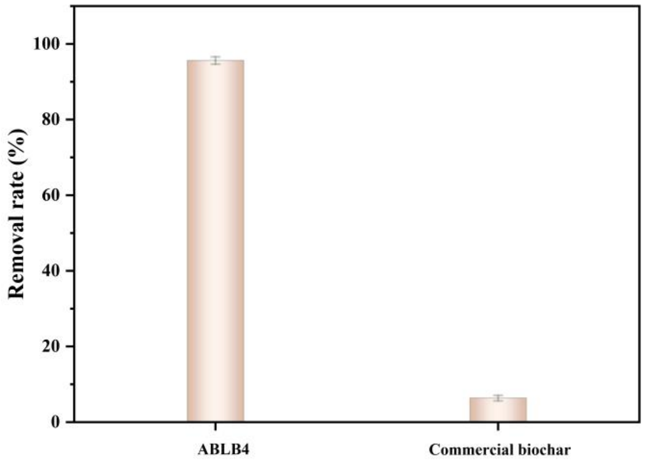


**Figure S3.** NOR adsorption effect comparison of ABLB4 and commercial biochar.

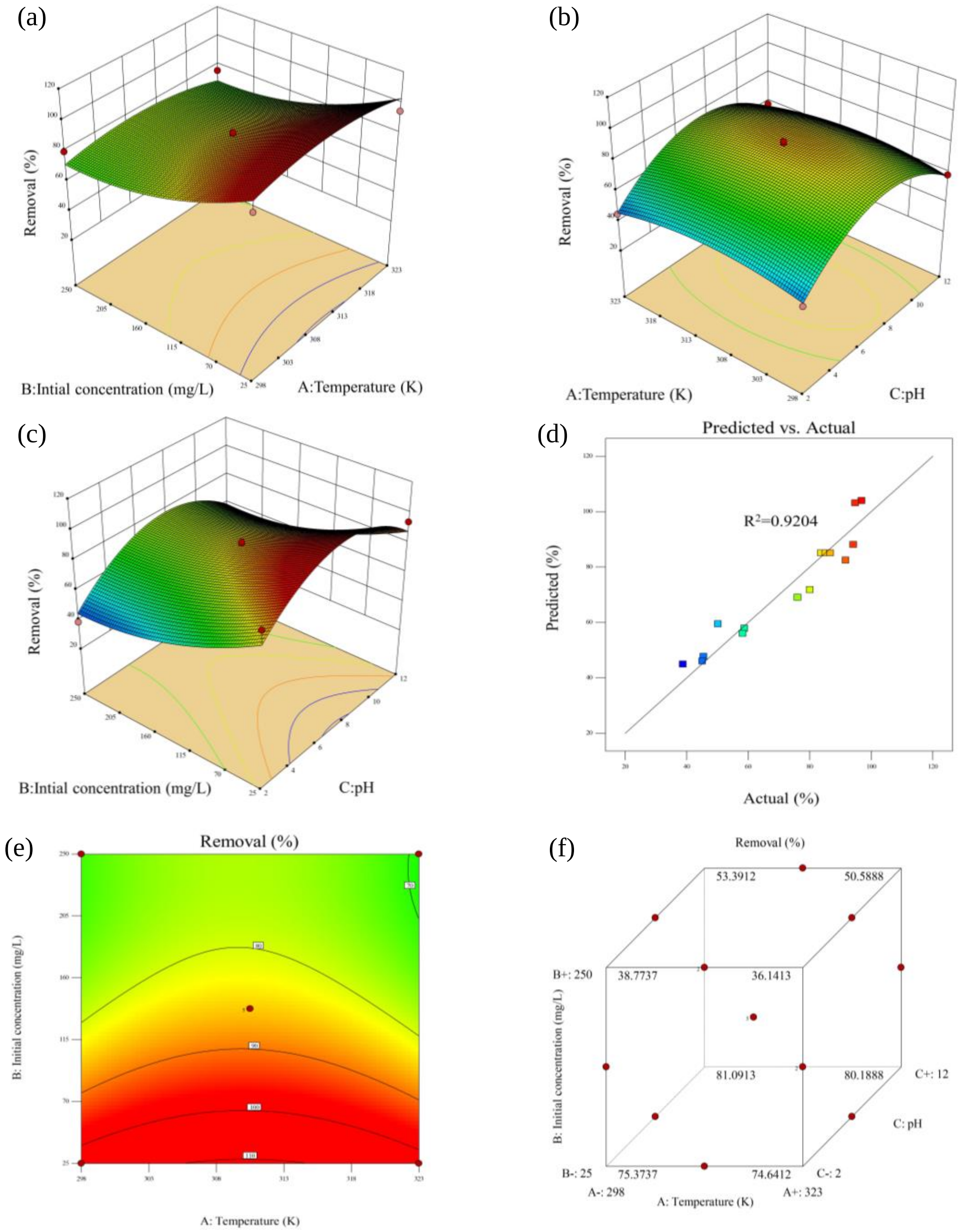


**Figure S4.** (a)-(c) Optimization of the NOR removal rate using response surface methodology. (d) Comparison between the predicted value with the actual removal rate. (e)-(f) contour plot and cube optimization for NOR removal rate.

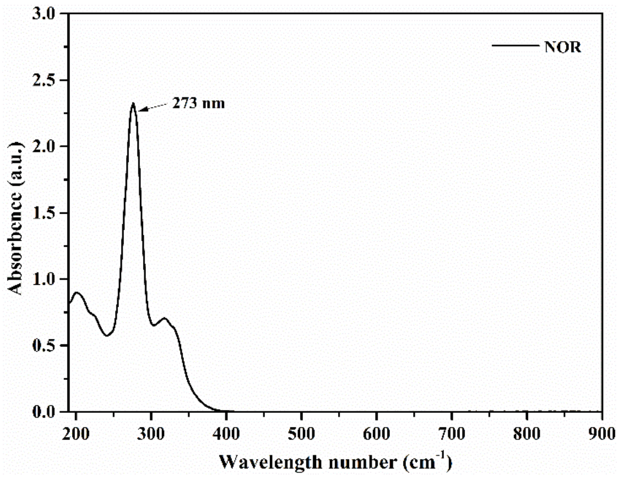


**Figure S5.** Full-wavelength scan of NOR.

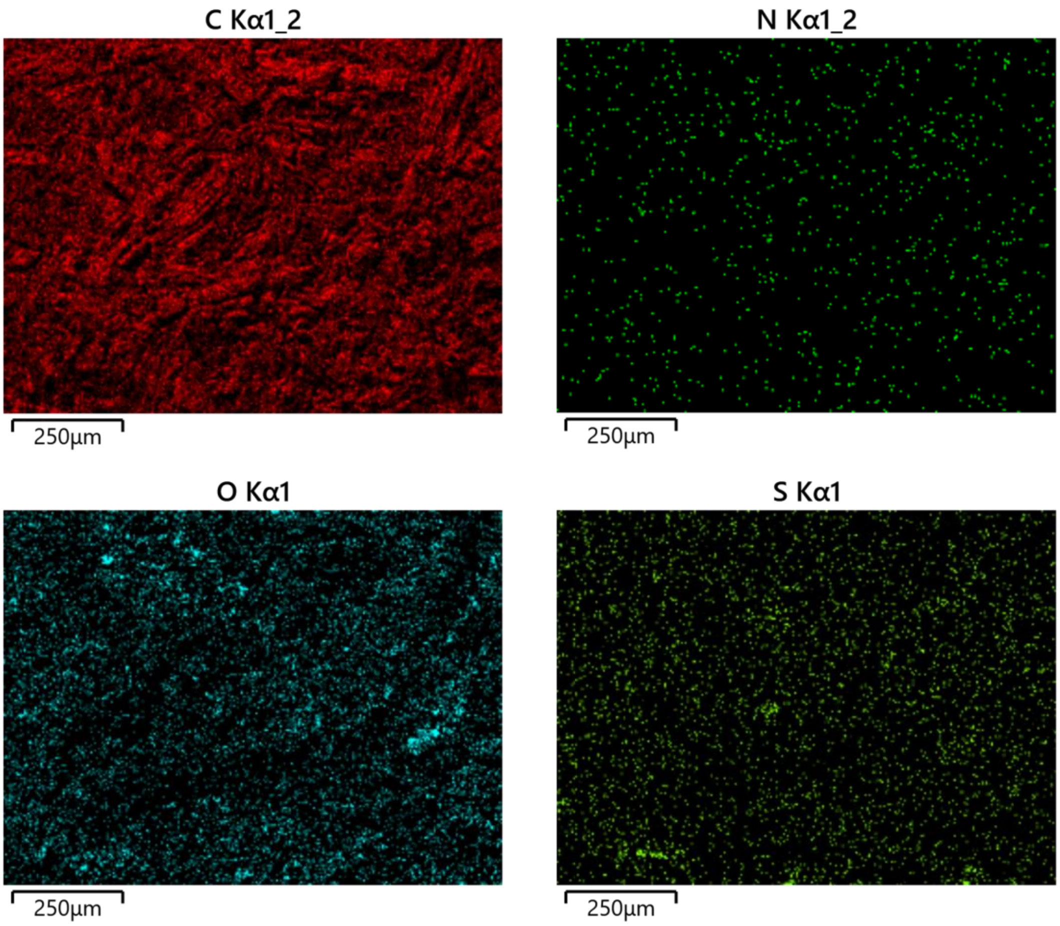


**Figure S6.** SEM-EDX microphotographs of ABLB4.

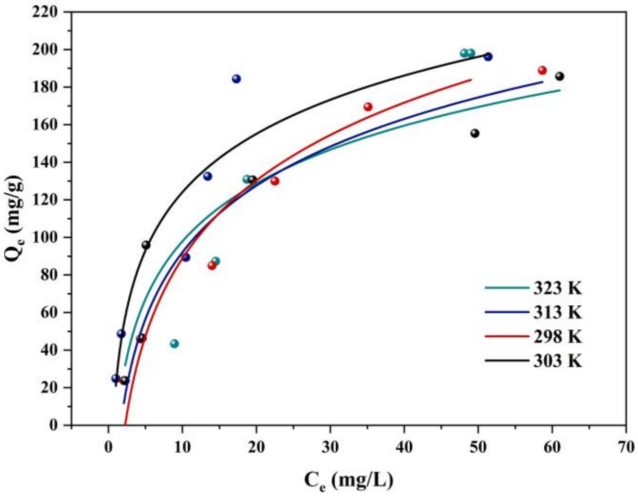


**Figure S7.** Temkin isotherm of ABLB4.

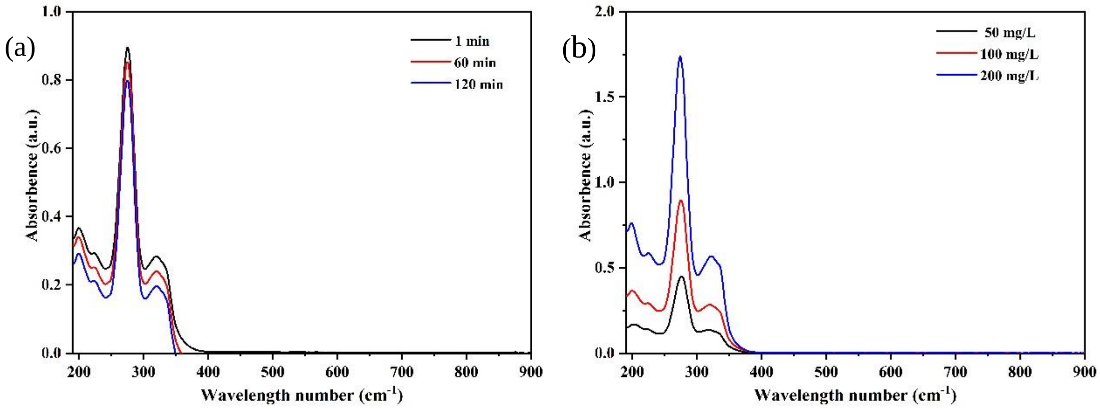


**Figure S8.** (a) UV full-wavelength scanning spectra of NOR with time. (b) UV full wavelength scanning spectra of NOR at different concentration.

**Table S1**

Detailed information of involved formulas and models.

| Formulas and models | Equation | Parameters |
|---|---|---|
| Adsorption capacity | $q_e = \frac{(C_0\text{-}C_e)V}{m}$ | $q_e$ (mg/g) are the adsorbed amount at an equilibrium concentration ($C_e$, mg/g)<br>$C_0$ (mg/L): initial NOR concentration<br>$C_e$ (mg/L): equilibrium NOR concentration<br>*V (L):* reaction solution volume of NOR<br>m (g): the sorbent mass |
| Removal rate | $E = \frac{(C_0\text{-}C_e)}{C_0}$ | $C_0$ (mg/L): initial NOR concentration<br>$C_e$ (mg/L): equilibrium NOR concentration |
| Pseudo-first order | $q_t = q_e(1 - e^{-k_1 t})$ | $q_e$ and $q_t$ (mg/g) are the adsorbed amount at an equilibrium concentration ($C_e$, mg/g) and a predetermined time ($t$, min)<br>$k_1$ ($min^{-1}$) is the rate constants for the PFO model |
| Pseudo-second order | $q_t = \frac{k_2 q_e^2 t}{1 + k_2 q_e t}$ | $k_2$ (g/mg min) is the rate constants for the PSO model |
| Weber-Morris intraparticle diffusion | $q_t = K_i t^{0.5} + C_i$ | Ki (mg/ (g·$min^{0.5}$)) is the adsorption rate constants of intra-particle diffusion model and Ci is the constant for film thickness of the intra-particle diffusion model. |
| Langmuir | $q_e = \frac{q_{max} K_L C_e}{1 + K_L C_e}$ | $q_{max}$ (mg/g) is the maximum adsorption capacity of the adsorbent<br>$q_e$ (mg/g) is the adsorption capacity at equilibrium<br>$C_e$ (mg/L) is the adsorbate concentration at equilibrium<br>$K_L$ (L/mg) is the constant for the affinity between the adsorbate and the adsorbent |
| Freundlich | $q_e = K_F C_e^{1/n}$ | $K_F$ (mg/g)/$(mg/L)^n$ is the Freundlich constant<br>*n* is a dimensionless Freundlich intensity parameter |
| Van der Hoff equation | $\Delta G = -RT \ln K_d$<br>$\ln K_d = \frac{\Delta S}{R} - \frac{\Delta H}{RT}$ | *ΔG, ΔH* and *ΔS* are the Gibbs energy (KJ/mol), activated enthalpy (KJ/mol) and the change in entropy (J/mol K), respectively;<br>$K_d$ is the distribution coefficient;<br>*R* is the ideal gas constant, whose value is 8.314 J /mol· K;<br>*T* is absolute temperature (K). |

**Table S2**

Kinetic constants of pseudo-first-order and pseudo-second-order models.

| Temperature | $q_{e,exp}$ (mg/g) | Pseudo-first-order | | | Pseudo-second-order | | |
|---|---|---|---|---|---|---|---|
| | | $K_1$ (min$^{-1}$) | $q_e$ (mg/g) | $R^2$ | $K_2$ (min$^{-1}$) | $q_e$ (mg/g) | $R^2$ |
| 298 K | 188.54 | 0.2005 | 189.80 | 0.9984 | 0.00094 | 228.73 | 0.9967 |
| 303 K | 181.36 | 0.1908 | 183.23 | 0.9923 | 0.00119 | 213.93 | 0.9858 |
| 313 K | 169.58 | 0.2366 | 169.96 | 0.9830 | 0.00219 | 188.42 | 0.9826 |
| 323 K | 155.39 | 0.3924 | 153.45 | 0.9888 | 0.00333 | 170.41 | 0.9844 |

**Table S3**

Weber-Morris intraparticle diffusion model of NOR adsorption by ABLB4.

| Temperature | First linear segment | | | Second linear segment | | | Third linear segment | | |
|---|---|---|---|---|---|---|---|---|---|
| | $K_{id}$ (mg/g/ min$^{0.5}$) | $C_i$ (mg/g) | $R^2$ | $K_{id}$ (mg/g/ min$^{0.5}$) | $C_i$ (mg/g) | $R^2$ | $K_{id}$ (mg/g/ min$^{0.5}$) | $C_i$ (mg/g) | $R^2$ |
| 298 K | 72.00 | 35.80 | 0.9979 | 16.45 | 112.30 | 0.9839 | 5.14 | 163.80 | 0.7750 |
| 303 K | 56.57 | 13.18 | 0.9904 | 19.41 | 95.55 | 0.9310 | 4.07 | 161.68 | 0.8311 |
| 313 K | 66.52 | 18.72 | 0.9693 | 27.73 | 62.60 | 0.9786 | 5.76 | 142.14 | 0.7311 |
| 323 K | 57.32 | 5.38 | 0.9907 | 14.44 | 100.63 | 0.9977 | 3.26 | 140.24 | 0.7790 |

**Table S4**

Lists of maximum NOR adsorption capacity on various biochar and other adsorption materials.

| Raw material | BET (m$^2$/g) | Total pore volume (cm$^3$/g) | Adsorbent dosage (g/L) | NOR concentration (mg/L) | $q_e$ (mg/g) | Ref. |
|---|---|---|---|---|---|---|
| Luffa sponge | 822 | 0.59 | 0.5 | 50-200 | 250 | [1] |
| Salix mongolica | 1348 | - | 2 | 25-266 | 210 | [2] |
| Porous resins and carbon nanotube | 160 | - | 5 | 20-100 | 154 | [3] |
| Spent coffee grounds | 46 | - | 1 | 10-50 | 70 | [4] |
| Cauliflower roots biochar | 232 | - | 2 | 10-300 | 31 | [5] |
| Pristine potato biochar | 90 | 0.12 | 4 | 4-20 | 5 | [6] |
| *Atropa belladonna* L. residue | 812 | 0.49 | 1 | 25-250 | 251 | This study |

**Table S5**

Thermodynamic parameters for NOR adsorption.

| Material | $\Delta G$ (kJ/mol) | | | | $\Delta H$ (kJ/mol) | $\Delta S$ (J/mol/K) |
|---|---|---|---|---|---|---|
| | 298 K | 303 K | 313 K | 323 K | | |
| ABLB4 | -6.44 | -6.20 | -5.29 | -3.27 | -43.79 | -124.45 |

**Table S6**

Energy consumption of biochar used in industrial wastewater production ($/t).

| Sample | Raw material | Disposal cost | Energy consumption | Wages of workers | Total cost | Pay back |
|---|---|---|---|---|---|---|
| ABLB4 | 1.5 | 15.3 | 178.2 | 140.5 | 335.5 | 852.5 |
| Commercial biochar | 311.6 | 28.3 | 518.1 | 330 | 1188 | |

**Table S7**

Elemental composition of raw ABL residues.

| Sample | C（%） | H（%） | O（%） | N（%） | S（%） |
|---|---|---|---|---|---|
| ABL residue | 46.13 | 4.96 | 42.26 | 1.78 | 0.16 |

**Table S8**

ICP-OES analysis of raw ABL residues.

| Sample | Si | Ca | Mg | K | Fe | Al | Na |
|---|---|---|---|---|---|---|---|
| ABL residues | 0.016 | 0.35 | 0.19 | 0.47 | 0.066 | 0.037 | 0.027 |

## References


1. Feng, Y.; Liu, Q.; Yu, Y.; Kong, Q.; Zhou, L.-l.; Du, Y.-d.; Wang, X.-f., Norfloxacin removal from aqueous solution using biochar derived from *luffa* sponge. *Journal of Water Supply: Research and Technology-Aqua* **2018,** 67, (8), 703-714. http://doi.org/10.2166/aqua.2018.040.
2. Liu, P.; Li, H.; Liu, X.; Wan, Y.; Han, X.; Zou, W., Preparation of magnetic biochar obtained from one-step pyrolysis of salix mongolica and investigation into adsorption behavior of sulfadimidine sodium and norfloxacin in aqueous solution. *J. Dispersion Sci. Technol.* **2019,** 41, (2), 214-226. http://doi.org/10.1080/01932691.2018.1562354.
3. Yang, W.; Lu, Y.; Zheng, F.; Xue, X.; Li, N.; Liu, D., Adsorption behavior and mechanisms of norfloxacin onto porous resins and carbon nanotube. *Chem. Eng. J.* **2012,** 179, 112-118. http://doi.org/10.1016/j.cej.2011.10.068.
4. Nguyen, V. T.; Vo, T. D.; Nguyen, T. B.; Dat, N. D.; Huu, B. T.; Nguyen, X. C.; Tran, T.; Le, T. N.; Duong, T. G.; Bui, M. H.; Dong, C. D.; Bui, X. T., Adsorption of norfloxacin from aqueous solution on biochar derived from spent coffee ground: Master variables and response surface method optimized adsorption process. *Chemosphere* **2022,** 288, (2), 132577. http://doi.org/10.1016/j.chemosphere.2021.132577.
5. Qin, T.; Wang, Z.; Xie, X.; Xie, C.; Zhu, J.; Li, Y., A novel biochar derived from cauliflower (*Brassica oleracea* L.) roots could remove norfloxacin and chlortetracycline efficiently. *Water Sci. Technol.* **2017,** 76, (11-12), 3307-3318. http://doi.org/10.2166/wst.2017.494.
6. Li, Y.; Wang, Z.; Xie, X.; Zhu, J.; Li, R.; Qin, T., Removal of Norfloxacin from aqueous solution by clay-biochar composite prepared from potato stem and natural attapulgite. *Colloids Surf. Physicochem. Eng. Aspects* **2017,** 514, 126-136. http://doi.org/10.1016/j.colsurfa.2016.11.064.
